# An analytical expression for R50% dependent on PTV surface area and volume: a lung SBRT comparison

**DOI:** 10.1002/acm2.13026

**Published:** 2020-09-30

**Authors:** Dharmin D. Desai, E. L. Johnson, Ivan L. Cordrey

**Affiliations:** ^1^ Department of Radiation Oncology CHI Memorial Hospital Chattanooga TN USA; ^2^ Department of Radiation Medicine University of Kentucky Chandler Medical Center Lexington KY USA

**Keywords:** analytic equation, lung SBRT, PTV surface area, R50%

## Abstract

In stereotactic body radiation therapy (SBRT), R50% is a common metric for intermediate dose spill and is defined in RTOG 0915 as the ratio of 50% isodose cloud volume (IDC50%) to the planning target volume (PTV). By coupling sound physical principles with the basic definition of intermediate dose spill, we derive an exact analytical expression for R50% for the case of a spherical volume. This expression for R50% depends on three quantities: the surface area of PTV (SA_PTV_), the volume of PTV (V_PTV_), and the dose gradient Δr. Validity of our analytical expression for R50% was confirmed via direct comparison to peer‐reviewed, multi‐institutional, diverse clinical data. The comparison of our R50% values computed from our analytical expression to the clinical data yielded an average percent difference of 3.8 ± 4.5%.

## 1 INTRODUCTION

Steep dose gradients are a requirement in high dose per fraction, hypofractionated approaches such as lung SBRT or cranial SRS/SRT treatment planning. To achieve a steep dose gradient, it is important to minimize intermediate dose spill. According to RTOG 0915, intermediate dose spill is quantified by the ratio of the 50% prescription isodose cloud volume (V_IDC50%_) to the planning target volume (V_PTV_) and is commonly referred to as R50% as shown in Eq. (1).^1^

(1) $$R50\%=\frac{V_{\text{IDC}50\%}}{V_{\text{PTV}}}$$

Narayanasamy et al. and Hoffman et al. have retrospectively analyzed clinical data to characterize the intermediate dose spill and presented R50% in a functional form that varies with the volume of PTV.^2^, ^3^ There is considerable dispersion in R50% values about the line predicted by the data fit, especially at smaller PTV volumes, for example, fig. 3 in Hoffman et al.^3^ A study by Goldbaum et al. discussed this dispersion seen in R50% values obtained for nearly equal PTV volumes and attributed the phenomenon to the PTV shape.^4^ However, Goldbaum et al. were not able to successfully formulate a methodology that addressed the supposition that PTV shape plays a role in the value of R50% achievable in a treatment planning scenario.

We propose that the surface area of the PTV (SA_PTV_) links the PTV shape to V_PTV_ and plays a central role in limiting attainable values of R50%. Using the SA_PTV_ as a surrogate for PTV shape, we derived an analytic expression for R50% for the special case of a spherical PTV volume. Using this model, we generated values for R50% that were then compared to data available from Hoffman et al. The power law data fits developed by Hoffman et al. for R50% and gradient measure (GM) are dependent only on PTV volume and based on a very robust, comprehensive retrospective analysis of 374 lung SBRT cases from multiple institutions.^3^ An independent comparison to this peer‐reviewed, clinically relevant data is meaningful because it confirmed the validity and limits of our analytical R50% equation derived from first principles.

## 2 MATERIALS AND METHODS

### 2.A Analytical derivation of R50%

**Fig. 1:**
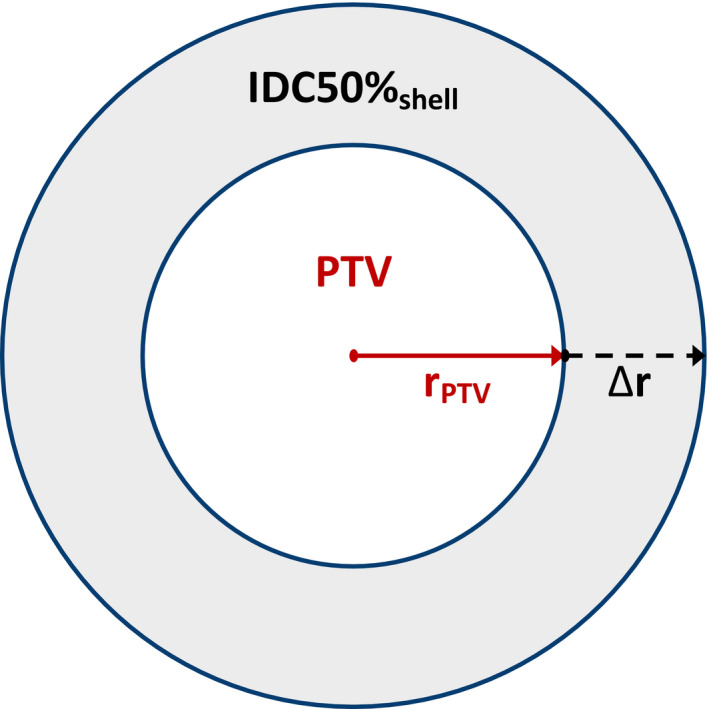
Plane through the center of the spherical volumes. Inner volume is the planning target volume (PTV). The shaded region is the spherical shell bounded by the 50% isodose cloud and the PTV surface area. ∆r is the radial thickness of the shell.

Consider a spherical PTV volume (V_PTV_) surrounded by a spherical shell that encloses the 50% isodose volume (IDC50%_shell_) as illustrated in Fig. 1. The sum of V_PTV_ and IDC50%_shell_ is the V_IDC50%_ given in Eq. (1).

By replacing V_IDC50%_ in Eq. (1) with the sum of the volumes, R50% becomes:

(2) $$R50\%=\frac{V_{\text{IDC}50\%}}{V_{\text{PTV}}}=\frac{V_{\text{PTV}}{\;+\;V}_{\text{IDC}50\%\text{shell}}}{V_{\text{PTV}}}=1+\frac{V_{\text{IDC}50\%\text{shell}}}{V_{\text{PTV}}}$$

Further, we determined an exact value of IDC50%_shell_ by integrating the spherical differential shell volume, 4πr^2^ dr, from r = r_PTV_ to r = r_PTV_ + ∆r:

(3) $$V_{\text{IDC}50{\%}_{\text{shell}}}=\int_{r_{\text{PTV}}}^{r_{\text{PTV}}+\Delta r}4\pi r^{2}\text{dr}\;=\;\frac{4}{3}\pi \left[{\left(r_{\text{PTV}}+\Delta r\right)}^{3}‐r_{\text{PTV}}^{3}\right]=4\pi r_{\text{PTV}}^{2}\Delta r\left(1+\frac{\Delta r}{r_{\text{PTV}}}+\frac{1}{3}{\left(\frac{\Delta r}{{\;r}_{\text{PTV}}}\right)}^{2}\right)$$

Given that SA_PTV_ = 4π(r_PTV_)^2^ and combining Eqs. (2) and (3), the resulting analytical form of R50% can be expressed as:

(4) $$\text{R50\%}{\;}_{\text{Analytic}}=1+\frac{{\text{SA}}_{\text{PTV}}}{V_{\text{PTV}}}\mathrm{\Delta r}\left[1+\left(\frac{\mathrm{\Delta r}}{r_{\text{PTV}}}\right)+\frac{1}{3}{\left(\frac{\mathrm{\Delta r}}{r_{\text{PTV}}}\right)}^{2}\right]$$

Equation (4) is the exact form of R50% for a spherical volume. We identified the three terms within the square brackets of Eq. (4) as zeroth‐order, first‐order, and second‐order terms, respectively, which is an extension of our previous work that only used the zeroth‐order term.^5^ When r_PTV_ is large compared to ∆r, the first‐ and second‐order terms are small compared to the zeroth‐order term 1. However, for small PTV volumes when r_PTV_ is comparable to ∆r, the first‐ and second‐order terms are significant, and utilizing the exact expression should improve the agreement for smaller PTV volumes over that seen in our previous study.^5^ It must be noted that an analytical expression for ∆r is not available in this model and additional treatment planning information must be utilized to estimate this parameter.

### 2.B Comparison methodology

Hoffman et al. provided clinical data in binned form for R50% and GM with respect to V_PTV_.^3^ The VMAT plans used in the Hoffman et al. study are highly conformal with the majority of cases having a conformity index ≤ 1.05. For these types of highly conformal plans, the 100% isodose volume that forms the basis of the GM spatially coincides with the PTV volume. Therefore, in such cases it is reasonable to assume ∆r is essentially equivalent to GM. Using the binned clinical GM values for ∆r and the assumed spherical geometry, we determined R50%_Analytic_ values using Eq. (4) for the V_PTV_ values given by Hoffman et al. The R50%_Analytic_ values were then directly compared to the clinical R50% values (R50%_Clinical_) given in table 1 of Hoffman et al.

## 3 RESULTS

**Fig. 2:**
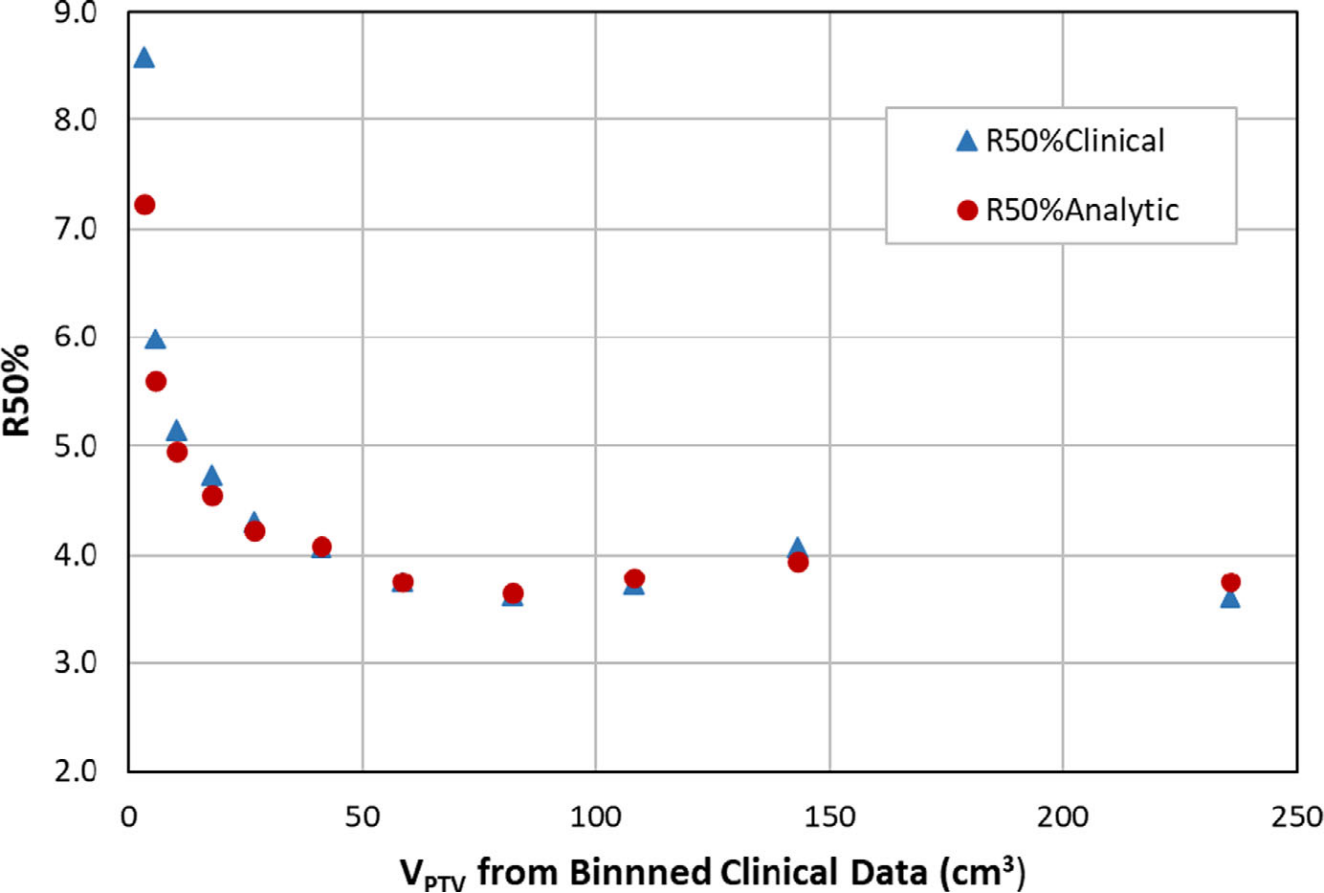
R50% vs V_PTV_ comparison of binned clinical R50% values and R50%_Analytic_ values. Triangles are R50%_Clinical_ values from Table 1 of Hoffman et al. Circles are R50%_Analytic_ values generated from Eq. (4).

Values of R50%_Analytic_ for a set of PTV volumes consistent with the work of Hoffman et al. are summarized in Table 1 and shown graphically in Fig. 2. Good agreement of R50%_Analytic_ values with the R50%_Clinical_ values can be seen in this comparison. The percent difference (%Diff) in these data are larger for the smaller PTV volumes but improve significantly for larger PTVs. The %Diff ranges from 15.9% at 3.1 cm^3^ to 0.2% for the 58.4 cm^3^ PTV. The average %Diff and standard deviation are 3.8% and 4.5%, respectively. The clinical data at very low and very high volumes are sparse, and comparisons at these volumes may suffer from statistical limitations.

**Table 1:** Comparison of R50%_Analytic_ values to clinical data of Hoffman et al.

V_PTV_ (cm^3^)^a^	r_PTV_ (cm)^b^	SA_PTV_ (cm^2^)^b^	GM (cm)^a^	R50%_Clinical_ ^a^	R50%_Analytic_	%Diff
3.05	0.90	10.17	0.84	8.59	7.23	15.85
5.72	1.11	15.46	0.86	5.99	5.59	6.63
9.93	1.33	22.34	0.94	5.14	4.96	3.59
17.55	1.61	32.65	1.06	4.73	4.55	3.74
26.53	1.85	43.01	1.14	4.30	4.22	1.85
41.11	2.14	57.59	1.28	4.07	4.08	0.21
58.35	2.41	72.74	1.33	3.75	3.74	0.18
81.93	2.69	91.21	1.45	3.62	3.64	0.51
108.08	2.96	109.71	1.65	3.72	3.78	1.72
143.05	3.24	132.25	1.88	4.07	3.94	3.21
235.67	3.83	184.48	2.12	3.61	3.75	3.79
					Ave %Diff	3.75
					Std Dev	4.45

^a^ Data from table 1 in Hoffman et al.

^b^ Parameters determined by the assumed spherical PTV shape. R50%_Analytic_ values were calculated from Eq. (4) using the given binned V_PTV_, SA_PTV_, and GM values.

## 4 DISCUSSION

For simplicity, the derivation of Eq. (4) assumed a spherical PTV and dose gradients that are isotropic around the PTV suggestive of 4π beam geometry. Isotropic dose gradients in lung SBRT cases are not achievable with realistic treatment delivery technology given the limitations in radiation beam positioning with these systems. For example, conventional C arm LINACs only are capable of coplanar delivery along the circumference of a circle when considering only the Gantry rotation. Some non‐coplanar character is possible when introducing rotations of the patient Couch support. However, consideration of the body habitus and PTV location limits the range of allowable Gantry and Couch positions resulting in some subset of 4π beam geometry available for treatment delivery. In addition, consideration of the relative locations of normal structures which are to be spared to the degree possible further complicates beam delivery geometry. Consequently, clinically relevant delivery geometry would not be expected to yield an isotropic dose drop‐off around the PTV. Typical SBRT lung treatments yield dose gradients in the axial direction that are larger than those obtained along the longitudinal patient axis. Our assumption regarding isotropic dose gradient is potentially problematic. However, it has been suggested that due to the conservation of integral dose, volumes of V_IDC50%_ will be approximately similar with either ideal or nonideal delivery geometries.^6^ Therefore, we expect only a weak dependence on treatment delivery technique, that is, VMAT versus static‐gantry IMRT. Nevertheless, validation against patient data is of paramount importance. The data of Hoffman et al. provided a robust clinical data set consisting of 374 actual patient plans covering a broad range of PTV volumes and locations and obtained from multiple institutions. Figure 2 shows remarkably good agreement between R50%_Analytic_ and R50%_Clinical_ suggesting our model limitations are not overly simplistic.

As seen in Fig. 2 and Table 1, disagreement between calculated R50%_Analytic_ values and R50%_Clinical_ values are larger at lower PTV volumes. Potential reasons for the increased disagreement for smaller volumes include the sensitivity of Eq. (4) to uncertainties in Δr and r_PTV_ for small PTV volumes (as previously discussed in the METHODS section), limitations of discretization of small volume shapes in treatment planning, limitations of the MLC leaf width when conforming to small volumes, and comparison to an average value of R50% in the data of Hoffman et al. having large spread in values. We believe the latter is an important point to consider for our model as the dispersion of R50% values about a given V_PTV_ cannot be explained using V_PTV_ alone. Our model that also considers the PTV shape through the SA_PTV_ and can allow for multiple predictions for R50% for a given V_PTV_. The dispersion in R50% values seen in clinical data can be reproduced, at least to some degree, by Eq. (4). Therefore, this model could potentially provide an improved estimate of the optimum R50% achievable in a given treatment planning scenario than a model that only considers V_PTV_.

The fitting of experimental data to algebraic expressions are phenomenological by nature and often obscures the fundamental physical nature of the processes that leads to this data. The approach used by Hoffman et al. was to fit experimental data of R50% and GM to a power law relation.^3^ While this approach provides a useful predictive tool for the treatment planner, it nonetheless does so without an explicit basis in the fundamental physics of R50% or GM. Our derivation of R50%_Analytic_ is based on physical principles and directly incorporates knowledge of the PTV characteristics (SA_PTV_, V_PTV_) and the dose falloff gradient. Since the PTVs in this study are assumed to be spherical, required information for the surface area and radius are easily determined from the known characteristics of a sphere. However, the dose gradient Δr is an unknown parameter that must be evaluated from additional information. Given the comparison of R50% we make to the data of Hoffman et al.,^3^ it is reasonable to use the GM values obtained from their publication as an estimate of Δr. Our equation for R50%_Analytic_ [Eq. (4)] provides new insights into the behavior of R50%, especially for small volumes where, as the PTV volume decreases, its effective radius also decreases. For these smaller PTVs, the steep rise in R50% is due to the increase in surface area to volume ratio (SA_PTV_/V_PTV_), and the first and second‐order terms of (Δr/r_PTV_) begin to play a dominant role in determination of R50%. We believe we have presented a more complete representation of R50% in its dependence on SA_PTV_, V_PTV_, and the dose gradient measure ∆r.

A PTV of a given volume can manifest several surface areas depending on the shape. It was hypothesized by Goldbaum et al. that an increase of the 12 Gy volume in Cranial SRS could be related to the increase in surface area of the target; however, they were not successful in quantifying the effect of surface area on this supposition.^6^ Our analytically derived relationship of R50% shows that this parameter is not uniquely defined by the PTV volume and its corresponding value of ∆r. A range of R50% values are possible depending on the SA_PTV_ and the corresponding PTV shape. Considering fig. 3 from Hoffman et al., we see that for a given value of PTV = 20 cm^3^, R50% varies between approximately 3.6 and 5.4. Such variability in the clinical value of R50% can be attributed to many factors: planner variability, location of PTV within the lung, tissue density heterogeneity within the PTV, locations of organs at risk (OARs) with respect to PTV, shape of the PTV, etc. Here, the shape of the PTV is the direct manifestation of the surface area of the PTV. Having a more quantifiable understanding of the PTV surface area is important as we have shown in our previous work.^5^

The predictive capability of the analytical R50% equation can serve as a useful tool to guide the treatment planner when optimizing the R50% value and potentially reduce the optimization time. For example, consider two equal PTV volumes with one being spherical and the other spheroidal. As discussed above, the power law fit of Hoffman et al. would suggest the same R50% is attainable in both cases. However, given the fact that the surface area of the spheroid would be larger than that of the sphere, our analytical expression would predict a larger R50% for the spheroid. Knowing a priori the shape/surface area of the PTV and the limiting R50% value, the planner could potentially be spared the effort involved in pursuing an unattainable result.

## 5 CONCLUSION

An analytical expression for R50% was derived for the special case of spherical volumes. The expression agrees well with peer‐reviewed data for R50% from Hoffman et al. We surmise that the surface area of the PTV plays an important role in the determination of the R50% value ultimately achievable in treatment planning and that our analytical expression can rationally explain the dispersion in R50% values for a given volume of PTV. More research is needed to ascertain the role of PTV surface area in the determination of treatment planning outcomes.

## CONFLICT OF INTEREST

The authors declare no conflict of interest.
